# *Brucella* Species Circulating in Smallholder Dairy Cattle in Tanzania

**DOI:** 10.3390/pathogens13090815

**Published:** 2024-09-21

**Authors:** Isaac Joseph Mengele, James Miser Akoko, Gabriel Mkilema Shirima, Shedrack Festo Bwatota, Shabani Kiyabo Motto, Luis E. Hernandez-Castro, Daniel Mushumbusi Komwihangilo, Eliamoni Lyatuu, Barend Mark de Clare Bronsvoort, Elizabeth Anne Jessie Cook

**Affiliations:** 1 Department of Global Health and Biomedical Sciences, School of Life Science and Bioengineering, The Nelson Mandela African Institution of Science and Technology (NM-AIST), Arusha P.O. Box 447, Tanzania; gabriel.shirima@nm-aist.ac.tz (G.M.S.); bwatota.shedrack@yahoo.com (S.F.B.); skymotto@gmail.com (S.K.M.); 2 Tanzania Veterinary Laboratory Agency (TVLA), Central Zone Laboratory, Dodoma P.O. Box 543, Tanzania; 3 International Livestock Research Institute (ILRI), P.O. Box 30709, Nairobi 00100, Kenya; j.akoko@cgiar.org; 4 Centre for Tropical Livestock Genetics and Health (CTLGH), International Livestock Research Institute (ILRI), P.O. Box 30709, Nairobi 00100, Kenya; 5 Tanzania Veterinary Laboratory Agency (TVLA), Central Veterinary Laboratory, Dar Es Salaam P.O. Box 9254, Tanzania; 6 National Health Service (NHS) Greater Glasgow and Clyde, 1055 Great Western Road, Glasgow G12 0XH, UK; enriqhernandez18@gmail.com; 7 Tanzania Livestock Research Institute (TALIRI), Dodoma P.O. Box 834, Tanzania; danielkomwihangilo@gmail.com; 8 International Livestock Research Institute (ILRI), Dar Es Salaam P.O. Box 34441, Tanzania; e.lyatuu@cgiar.org; 9 The Epidemiology, Economics and Risk Assessment (EERA) Group, The Roslin Institute at the Royal (Dick) School of Veterinary Studies, University of Edinburgh, Midlothian EH25 9RG, UK; mark.bronsvoort@roslin.ed.ac.uk; 10 Centre for Tropical Livestock Genetics and Health (CTLGH), The Roslin Institute at the Royal (Dick) School of Veterinary Studies, University of Edinburgh, Midlothian EH25 9RG, UK

**Keywords:** dairy cattle, brucellosis, qPCR, molecular prevalence, *Brucella*, Tanzania

## Abstract

Brucellosis is a zoonosis caused by bacteria of the genus *Brucella*, which results in economic losses relating to livestock and threatens public health. A cross-sectional study was conducted to determine the molecular prevalence of *Brucella* species in smallholder dairy cattle in six regions of Tanzania from July 2019 to October 2020. Dairy cattle (*n* = 2048) were sampled from 1371 farms. DNA extracted from blood and vaginal swabs was tested for *Brucella* using qPCR targeting the IS711 gene and positives were tested for the alkB marker for *B. abortus* and BMEI1172 marker for *B. melitensis*. The molecular prevalence was 3.5% (95% CI: 2.8–4.4) with the highest prevalence 8.1% (95% CI: 4.6–13.0) in Njombe region. *B. melitensis* was the predominant species detected (66.2%). Further studies are recommended to understand the source of *B. melitensis* and its implications for veterinary public health. Livestock keepers should be informed of the risks and biosecurity practices to reduce the introduction and control of *Brucella*. Cattle and small ruminant vaccination programs could be implemented to control brucellosis in high-risk populations in the country.

## 1. Introduction

Brucellosis is a zoonotic bacterial disease that causes economic loss in dairy cattle production systems. Brucellosis is considered to be one of the most widespread zoonotic diseases globally [1]. The disease is caused by a bacterium of the genus *Brucella*. Of the twelve *Brucella* species that are known to affect mammals, the common species that affect domestic animals are *B. abortus* in cattle, *B. melitensis* in goats, *B. ovis* in sheep, *B. suis* in pigs and *B. canis* in dogs [2,3]. *Brucella* spp. are somewhat host-specific; however, recent studies have highlighted the importance of cross-species infection [4,5,6]. Studies have found that brucellosis in cattle can also be caused by *B. melitensis* or *B. suis* [2,7,8]. This renders eradication through vaccination with *B. abortus*-derived vaccines ineffective, since the efficacy of the S.19 vaccine, which is widely used in endemic areas, has not been fully validated against *B. melitensis*, and those vaccines which confer cross-protection may not be available, especially in low- and middle-income countries (LMICs) [9,10,11].

In Tanzania, the first isolates of *B. abortus* and *B. melitensis* from cattle and goats, respectively, were obtained in 1967 [12]; however, no typing of the isolates was performed [12]. In 2015, *B. abortus* was isolated from aborted materials of dairy cattle in Njombe region and the first typing identified *B. abortus* biovar 3 [13]. Around the same time, *B. abortus* biovar 1 was detected and typed from cow’s milk [14]. In recent years, mixed farming practices have been reported to be associated with brucellosis reemergence in Tanzania [15,16]. Research in neighboring countries has identified cattle infected with other *Brucella* species. Studies in Kenya, Uganda and Rwanda have identified *B. melitensis* and *B. abortus* in dairy cattle [6,8,17,18]. Furthermore, *B. melitensis*, the most pathogenic of the classical *Brucella* species, has been frequently isolated from febrile human patients in northern Tanzania [19,20]; however, the possible sources of infection in humans have yet to be established. The authors, concluded that to control human brucellosis, vaccination should also target small ruminants by using the *B. melitensis* REV1 vaccine [19]. In Tanzania, there have not been any reports of isolation or molecular detection of *B. melitensis* in cattle. Therefore, the objective of the current study was to identify *Brucella* species circulating in smallholder dairy cattle populations in Tanzania by using molecular techniques.

## 2. Materials and Methods

### 2.1. Study Area and Design

**Figure 1:**
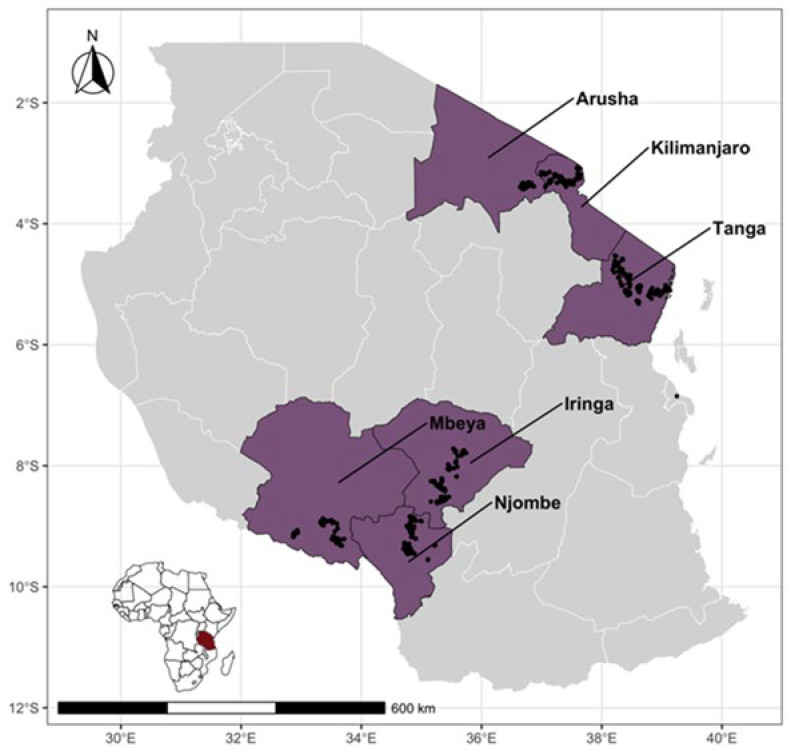
Map of Tanzania showing study regions (in purple) with large populations of smallholder dairy cattle and unstudied regions in gray (right). Black dots indicate the locations of the sampled cattle. The inset shows the location of Tanzania in Africa.

A cross-sectional study was conducted from July 2019 to October 2020 to identify the Brucella species circulating in smallholder dairy farming systems in two agroecological zones comprising six administrative regions of Tanzania. The study was conducted in three regions in the northern zone (Kilimanjaro, Arusha and Tanga), involving 252,554 dairy cattle, and three regions in the southern highland zone (Iringa, Njombe and Mbeya), involving 103,306 of dairy cattle (Figure 1). These regions have the highest density of smallholder dairy cattle in Tanzania [21,22]. The number of dairy cattle in each study region and the sample size estimation are elaborated upon in our previously published article [16]. According to the household budget survey of 2018, all six regions were above the food poverty line of TSH 33,748 (USD 13) per person per month [23]. All the study regions practice mixed farming, in which dairy cattle interact with other domestic animals [16].

### 2.2. Study Population

Dairy cattle kept under smallholder farming systems were the target of our study. The dairy cattle in these regions are mainly Friesian, Ayrshire and Jersey crossbreeds, with Tanzanian Short Horn Zebu (TSHZ) and Friesian crosses comprising the largest proportion (80%) of breeds. The feeding management systems of these dairy cattle were twofold, involving (1) an intensive management system in which pastures were cut and carried to the farm for them to feed on and (2) an extensive system in which cattle were left to graze on private or communal land. The dairy cattle in this study were selected from a subset of the dairy cattle registry of the Africa Dairy Genetics Gains (ADGGs) program (https://data.ilri.org/portal/dataset/adgg-tanzania, accessed on 1 June 2019). The ADGGs project randomly registered over 52,500 cattle across the study regions in the database. Furthermore, 4000 dairy cattle were randomly selected and genotyped [24]. The sample for this study was selected from the genotyped animals, although at the time of the study, not all the genotyped animals were available because of the high rate of animal removal from the farms due to the sale, natural death or slaughter and the time that elapsed between genotyping and sampling. The sample size estimation for a concurrent seroprevalence study was calculated with a seroprevalence estimate of 5% (with 3% precision) and 95% confidence interval for the smallest region, assuming simple random sampling [16].

### 2.3. Blood and Swab Sampling from Dairy Cattle and Samples Storage

A cross-sectional survey was conducted at 1371 farms. A total of 2049 dairy cattle were sampled, and 5 mL blood was collected aseptically by venipuncture into EDTA, as explained in a previous study [25]. The animal’s identification number, the date of collection and the field barcode were labeled on each tube. In the field, all samples were kept in a cool box containing ice packs and transported to the field laboratory for storage on a daily basis. Similarly, vaginal swabs were collected aseptically after the vulva was cleaned using a chlorhexidine-soaked paper towel. The vulva lips were opened with fingers and a long shaft swab was inserted per vaginum, and the mucosa was gently swabbed by rotating the swab shaft left and right while removing it. After its removal, the swab was then inserted in a cryovial tube containing 1 mL sterile phosphate-buffered saline (PBS) and squeezed on the tube wall while the solution was mixed. The shaft was then cut off and thrown in a waste disposal bin, leaving the swab tip in the PBS cryovial. The PBS cryovial was then labeled with the collection date, barcoded, and scanned into the ODK form. Both EDTA blood tubes and PBS swab tubes were stored at −20 °C in an upright position until they were transported to the Nelson Mandela African Institution of Science and Technology laboratory in Arusha for longer-term storage.

### 2.4. DNA Extraction from EDTA Blood and PBS Swabs

EDTA tubes containing blood and PBS tubes containing a swab were allowed to thaw at room temperature on a table. After thawing, each tube was briefly mixed by vortexing. Three hundred microliters (300 µL) of blood/PBS was aliquoted and placed in a sample autoplate of TANBeads^®^. Total genomic DNA extraction was performed by using a TANBeads^®^ Nucleic Acid Extraction Validation Kit (OptiPure Blood DNA Auto Plate) designed for use with the Maelstrom 9600 (Taiwan Advanced Nanotech Inc, Taoyuan City, Taiwan), a robotic system in ILRI laboratories in Nairobi, Kenya. The extraction kit was suitable for isolating DNA from whole blood, including deep-frozen blood, and it used the silicone dioxide layer coated on the magnetic beads. A 100 µL genomic DNA extract was provided. After the extraction, different random DNA samples were tested for quality and degradation by using a Nanodrop spectrophotometer and 1% agar rose gel electrophoresis, respectively (Supplementary Material S1).

### 2.5. Real-Time PCR for Brucella Genus Detection and Species Characterization

The QuantiStudio 5 qPCR machine (Applied Biosystems, Woodlands, Singapore) with the 96-well plate format and 0.2 mL block installed with QuantiStudio TM Design and Analysis software v1.5 was used for the analysis.

For Brucella genera detection, the DNA samples were tested for the presence of insertion sequence IS711 by using the IS711 primer pair and probe Table 1 in a uniplex assay. The qPCR conditions and reaction volumes used were adopted from Akoko et al. [5]. The following reaction volumes were prepared: 5 µL of ready-to-use Master Mix (Luna Universal Probe qPCR Master Mix, New England BioLabs, MA, USA), 1.25 µL of IS711 Primer and Probe mix (Macrogen, The Netherlands), 1.75 µL molecular grade water and a 3 µL DNA template. The total reaction-mix volume (cocktail) of 11 µL was then thoroughly mixed via gentle vortexing for 1 minute before the 96 wells PCR plate was inserted into the qPCR machine.

The thermo cycler conditions were set as they would be for pretreatment with Uracil-DNA Glycosylases (UDGs) at 50 °C for 2 min, followed by polymerase activation and DNA denaturation at 95 °C for 10 min, an amplification step at 95 °C for 15 s and 1 min of annealing at 60 °C for 42 cycles.

Positive DNA samples based on the IS711 gene marker (positive for the Brucella genus) were further characterized for *B. abortus* and *B. melitensis* using *alkB* and *BMEI1172* primers and a probe, respectively. 

The reaction volumes used for *B. abortus-* and *B. melitensis*-specific assays were 7.5 µL Master mix (Perfecta qPCR ToughMix UNG Low ROX), 0.75 µL primer and probe (species-specific), 1.75 µL molecular-grade water, and 5 µL of DNA template. The final reaction mix (cocktail) of 15 µL was thoroughly mixed by vortexing for 1 min before the 96-well PCR plate was inserted into the qPCR machine.

The PCR conditions for *Brucella* species-specific assays were as follows: the pretreatment stage with Uracil-N-Glycosylase (UNG-step) was set at 45 °C for 5 min to cleave all contaminated templates containing U bases, followed by DNA denaturation at 95 °C for 5 min, amplification at 95 °C for 15 s, and annealing at 60 °C for 30 s for a total of 42 cycles.

The positive controls for the *Brucella* strains, *B. melitensis* 16 M and *B. abortus* 544, were both sourced from the Friedrich-Loeffler Institute, Germany. For the negative controls, a mixture of RNAase-free molecular-grade water and Master mix was used. The assay efficiency statistics and limit of detection of the tenfold serial dilution of the reference materials for genus and species detection are provided in Supplementary Material S2.

### 2.6. Spatial Analysis

A spatial scan statistic was used to detect statistically significant spatial clusters of PCR-positive animals in the Njombe, Kilimanjaro and Arusha Regions only. Cluster analyses were performed using the SaTScan™ v10.1 software [26] with a Bernoulli model for binary events (i.e., PCR-positive/PCR-negative). SaTScan uses Monte Carlo hypothesis testing to obtain the *p*-values and SaTScan adjusts for the underlying spatial homogeneity of a background population. For each location and scanning-window size, the alternative hypothesis was that there was an elevated risk within the window compared with the risk outside the window, and a likelihood ratio test was performed. Multiple different window sizes were used, and the selected locations were the latitude/longitude for each animal with slight jittering to avoid more than one animal being at the same location. The window with the maximum likelihood was the most likely cluster, i.e., the cluster that was least likely to be due to chance. A *p*-value was assigned to this cluster. For this analysis, we used 9999 Monte Carlo replications, and a cluster was considered to be statistically significant if the *p*-value was <0.05. There was no adjustment for within-herd clusters.

### 2.7. Data Analysis for Calculation of Molecular Prevalence

Data analysis was carried out using R software (version 4.2.3) [28,29] with individual animals as primary sampling units and the district as the clustering unit rather than the herd. This was because so many herds were small and had only one animal sampled. The prevalence was estimated as the ratio of PCR positives (numerator) and the total number of animals tested (denominator) for overall and regional prevalences and the *binom.test* function was used to generate the 95% binomial confidence interval. The design-adjusted overall prevalence was estimated after the different sampling weights were incorporated into the estimation using the *svydesign*, *confint* and *svyby* functions in the survey package [30]. This allowed the stratified study design to be accounted for in the prevalence estimates.

## 3. Results

### 3.1. Description of Sampled Dairy Cattle

A total of 2049 dairy cattle were sampled from 1371 farms across the six study regions. The median herd size was two cattle. The majority of sampled cattle were female (97.2%). The predominant breed was SHZ–Friesian crosses (68.7%), with other breeds being SHZ–Ayrshire (20.8%), SHZ–Jersey (6.9%) and indigenous breeds (3.6%).

### 3.2. Brucellosis Molecular Prevalence of Dairy Cattle in Selected Regions of Tanzania

There were 35/2046 blood and 37/1893 swab samples that were Brucella genus-positive (Table 2). There was no agreement between sample types.

**Figure 2:**
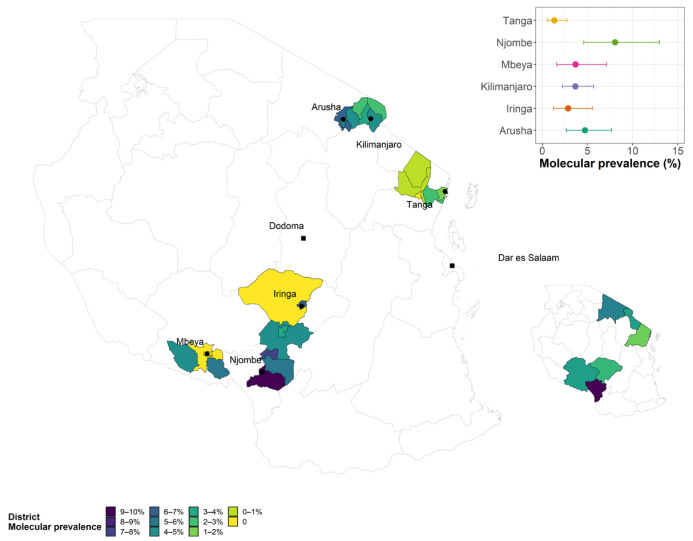
Choropleth map showing the regional molecular prevalence (insets) and the detailed molecular prevalence by local authority sampled in each region.

The overall unadjusted animal molecular (PCR) prevalence (with positivity determined either via a blood sample or via a vaginal swab) was 3.5% (95% CI: 2.8–4.4) and the overall design-adjusted prevalence was 3.7% (96% CI: 2.7–3.7). Among the study regions, Njombe region had the highest molecular (PCR) prevalence of 8.1%, followed by Arusha 4.7%, Mbeya 3.8% and Kilimanjaro region 3.7% (Table 3 and Figure 2).

### 3.3. Brucella Species Circulating in Dairy Cattle Population Identified from Brucella Genus-Positive Swab and Blood Samples

The majority of blood samples (19/35 (54.3%)) and vaginal swabs (29/37 (78.4%)) were PCR-positive for *B. melitensis* only, with a further 10/35 (28.6%) blood samples and 7/37 (18.9%) swabs found to be PCR-positive for both (*B. melitensis* and *B. abortus*), meaning that the vast majority of infections involved *B. melitensis* (Table 4). *B. abortus* occurred on its own in 2/35 (5.7%) blood samples. There were four blood samples (11.4%) and one swab sample (2.7%) in which no species was determined (Table 4).

### 3.4. Brucellosis Hotspot Areas

Figure 2 is a spatial choropleth map of Tanzania (main) with the right-bottom inset showing the study regions and their molecular prevalence, with the highest prevalence in Njombe region (dark blue), while the main map shows the molecular prevalence of the study districts (local authorities) for each study region. The main map shows that PCR-positive animals were clustered within a small number of local authorities in the Kilimanjaro, Mbeya, Arusha and Njombe regions.

### 3.5. Spatial Clustering of Brucella PCR-Positive Animals

**Figure 3:**
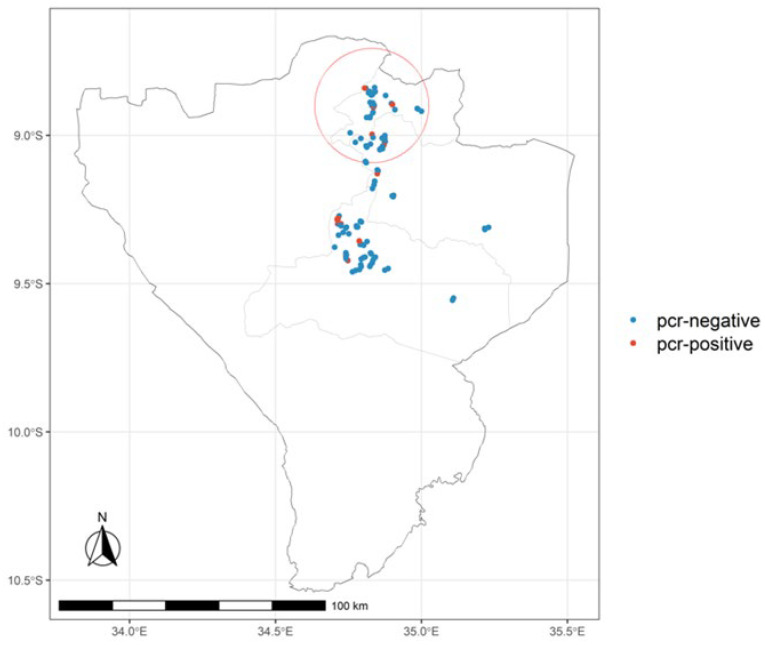
Map of Njombe region showing district boundaries, the location of PCR-positive and PCR-negative animals (jittered) and the radius (red circle) of the significant cluster identified by the SaTScan analysis.

To explore the spatial clustering pattern of animals, the Kilimanjaro, Arusha and Njombe regions were mapped to identify the cluster of PCR-positive animals. SatScan analysis identified five clusters in the three regions, but only one significant cluster was found in Njombe region, and therefore Njombe region was further mapped to have a closer view (Figure 3). In a cluster of five animals, four of them were PCR-positive with a relative risk of 16.98 in a radius of 1.36 km in northern Njombe region.

## 4. Discussion

Brucellosis is a globally neglected bacterial zoonosis. It was characterized for the first time in Tanzania, where it was identified in domestic animals in 1967 and again in 2015 [12,13,14]. In Tanzania, brucellosis in dairy cattle is endemic and has continued to affect dairy production and public health, apart from during a short period in the late 1990s when it was successfully controlled [31]. In Tanzania, most brucellosis studies have depended on the use of serological tests to provide recommendations and conclusions on the best way to control the disease in animals and have assumed *Brucella* host-specificity due to the lack of serological tools with which to differentiate them [32]. However, recent studies have shown that the host-specificity of *Brucella* species no longer applies, as cross-infections have recently been reported globally [6,33]. Therefore, molecular characterization of *Brucella* species is becoming increasingly important, as it will allow us to understand the differences in their epidemiology and make appropriate recommendations for control and eradication [34].

The current study reports the overall animal level-adjusted PCR prevalence (molecular prevalence) of 3.5% across the study regions, which represent the major dairy cattle-keeping areas of Tanzania, where roughly 50% of improved dairy cattle are located [21,22]. The molecular prevalence reported in this study is lower than the molecular prevalence of 18.9% reported in Kenya [5] and similar to the 5.6% prevalence reported in Rwanda [6]. The discrepancy in molecular prevalence is likely due to differences in the sample types used, sample size, study population and study locations, as the prevalence of brucellosis has been reported to be lower in highland areas in Kenya [35] which have a similar agroecology to the areas in this study. Clustering analysis revealed one significant cluster of molecular results analysis in northern part of Njombe region; a similar cluster was also revealed in serosurvey results in our previous analysis [16], suggesting that Njombe region is the brucellosis hotspot region that requires urgent interventions. To control the disease throughout the country, high-risk regions such as Njombe, Kilimanjaro, Mbeya and Arusha need to be prioritized for disease interventions [16].

The current study has identified that *B. abortus*, *B. melitensis* and undetermined *Brucella* species are circulating in this dairy cattle population. The study revealed that dairy cattle are predominantly PCR-positive for *B. melitensis*, which is generally considered to be a pathogen that affects sheep and goats. Our previous work has demonstrated an association between seropositivity and the presence of goats, with the odds of cattle being seropositive on a farm that keeps goats being 3.02 times greater than those odds for cattle on a farm that does not keep goats [16]. The role of goats in the epidemiology of cattle brucellosis in SSA has also been reported in previous studies [15]. It was not possible to revisit farms during this study to sample small ruminants to gain a better understanding of the epidemiology of *B. melitensis* in this setting. However, a focus on small ruminants is recommended for future studies.

The current study also reports dairy cattle PCR-positive for two *Brucella* species, *B. abortus* and *B. melitensis*, and undetermined *Brucella* species. Co-infections with more than one *Brucella* species have also been reported in Rwanda and in other African countries [6,33,36,37]. Furthermore, PCR-positivity for two *Brucella* species was attributed to the mingling of cattle and small ruminants [38,39]. The detection of an undetermined *Brucella* species highlights the potential for infection with other *Brucella* species such as *B. canis*, and *B. suis*, which have been identified in dairy cattle following natural infection in different countries [40,41,42] and may be related to the presence of other domestic animals such as sheep, dogs and pigs in dairy farms.

The presence of *Brucella* in vaginal swabs suggests that bacterial transmission may occur among dairy cattle in a herd and between herds as a result of contaminated drinking water and pasture [43]. The presence of *Brucella* in vaginal swabs of cattle has also been revealed by other studies [44,45]. Furthermore, the presence of *Brucella* in the vagina signifies the necessity of veterinarians, veterinary assistants and farmers using personal protective equipment when managing difficult calvings and retained placenta in cows.

The presence of *B. melitensis* and mixed *B. abortus* and *B. melitensis* PCR-positives in dairy cattle pose a challenge when it comes to controlling the disease by vaccination [46,47]. The monovalent *B. abortus* S19 vaccine which is produced in Tanzania may not be effective in controlling the disease under these scenarios (*B. melitensis* and co-infections), as the vaccine has not been fully validated for conferring cross-protection and alternative vaccines which may confer protection may not be available for use in LMICs [9,10,11]. Further validation of the currently available vaccines is required; furthermore, production of a bivalent vaccine (containing *B. abortus* and *B. melitensis*) might assist with the control and eradication of the disease in cattle [48,49].

This study had limitations, as there was no agreement regarding PCR positivity between vaginal swabs and blood from the same animal, which could be attributed to the short and transient bacteremia in cattle and the fact that shedding of bacteria in vaginal samples tends to occur post-calving.

Furthermore, the poor agreement could have been attributed to the long-term storage of samples, which were kept in a deep freezer at a very low temperature (−20 °C) for over a year; these conditions are likely to have degraded the samples. Poor agreement was also observed between the PCR and cELISA results published in our previous article [16], which could have been attributed to individual animals’ varying bacteremic and immunologic phases and abortion status. This finding is similar to other studies in cattle which found poor agreement between serological and PCR results in different samples from the same animal [50]; however, there was a higher chance of PCR positivity among animals with a history of abortion [51]. Therefore, to provide the highest disease detection rate, it is important to run the serological and molecular detection methods using different samples and therefore reduce the number of false-negative results.

The exclusion of serologic results previously reported [16] in this study may have led to a misclassification of animals with chronic *B. melitensis* infections and is likely to have resulted in an underestimation of the number of cattle affected. Finally, there was insufficient DNA to allow sequencing for further characterization of the pathogens.

## 5. Conclusions

The current study confirms that bacteria of the genus *Brucella* are circulating in smallholder dairy cattle, suggesting that brucellosis is present and is likely to be causing clinical disease in dairy cattle. The importance of *B. melitensis* infections in smallholder dairy cattle is not clear, and further understanding of the clinical significance and veterinary public health implications of these infections is needed.

This study recommends that further studies be conducted on *Brucella* species circulating in dairy cattle, and further studies on the roles of small ruminants and other domestic animals in the epidemiology of brucellosis in dairy cattle should also be carried out. Training famers in good biosecurity and control methods is recommended, as is vaccination of dairy cattle and small ruminants in high-risk populations.

## Figures and Tables

**Table 1:** Oligonucleotide primers and probes used to perform qPCR assays.

Target	Targeted Gene	Sequences of Primers and Probes (5′–3′)	Fluorophore/Quencher	Reference
Genus *Brucella*	IS711	Probe: AAG CCA ACA CCC GGC Forward: GGC CTA CCG CTG CGA AT Reverse: TTG CGG ACA GTC ACC ATA ATG	FAM/-MGBNFQ	Matero et al. (2011) [26]
*B. melitensis*	IS711 downstream of BMEI1162	Probe: CAGGAGTGTTTCGGCTCAGAATAATCCACA Forward: AACAAGCGGCACCCCTAAAA Reverse: CATGCGCTATGATCTGGTTACG	Texas Red/BHQ2	Probert et al. (2004) [27]
*B. abortus*	IS711 downstream of alkB	Probe: CGCTCATGCTCGCCAGACTTCAATG Forward: GCGGCTTTTCTATCACGGTATTC Reverse: CATGCGCTATGATCTGGTTACG	JOE/BHQ1	

**Table 2:** Regional distribution of Brucella genus positive blood and swab DNA samples from individual cattle in Tanzania.

Region	Total Animals Sampled	Number of Positive Blood Samples (%)	Number of Positive Swab Samples (%)
Arusha	318	5/318 (1.6%)	10/294 (3.4%)
Tanga	524	6/524 (1.0%)	1/412 (0.2%)
Kilimanjaro	521	11/519 (2.1%)	8/513 (1.6%)
Iringa	281	7/281 (2.5%)	1/273 (0.4%)
Njombe	187	1/186 (1.1%)	14/186 (7.5%)
Mbeya	218	5/218 (2.3%)	3/215 (1.4%)
Total	2049	35/2046 (1.7%)	37/1893 (2.0%)

**Table 3:** The combined molecular (PCR) prevalence based on Brucella genus-positive results.

Region	Negative	Positive	Total	PCR Prevalence %	95% CI	Dairy Cattle Population
Arusha	303	15	318	4.7	2.7–7.7	78,637
Tanga	517	7	524	1.3	0.5–2.7	41,639
Kilimanjaro	500	19	519	3.7	2.2–5.7	161,984
Iringa	273	8	281	2.8	1.2–5.5	7081
Njombe	171	15	186	8.1	4.6–13.0	7177
Mbeya	210	8	218	3.8	1.7–7.4	72,724
Total	1974	72	2046	3.5	2.8–4.4	369,242

**Table 4:** Real-time polymerase chain reaction (qPCR) results for *Brucella* species identified from genus-positive swab and blood samples.

Region	Sample	*B. abortus*	*B. melitensis*	Mixed	Undetermined
Arusha	Blood *n* = 5	0	3	2	0
	Swabs *n* = 10	0	9	1	0
Kilimanjaro	Blood *n* = 11	1	9	0	1
	Swabs *n* = 8	0	6	2	0
Tanga	Blood *n* = 6	0	2	3	1
	Swabs *n* = 1	0	1	0	0
Njombe	Blood *n* = 1	0	1	0	0
	Swabs *n* = 14	0	11	2	1
Iringa	Blood *n* = 7	0	3	2	2
	Swabs *n* = 1	0	1	0	0
Mbeya	Blood *n* = 5	1	1	3	0
	Swabs *n* = 3	0	1	2	0
Total	Blood *n* = 35	2	19	10	4
	Swabs *n* = 37	0	29	7	1

Mixed = PCR-positive for both *B. abortus* and *B. melitensis*.

## Data Availability

All relevant data are presented within the manuscript.

## Supplementary Materials

The following supporting information can be downloaded at https://www.mdpi.com/article/10.3390/pathogens13090815/s1, Supplementary Material S1: Genomic DNA quality checks; Supplementary Material S2: Statistics for the efficiency of PCR assays.
