# Supplementary material for: Brucella Species Circulating in Smallholder Dairy Cattle in Tanzania
Source: Pathogens. 2024 Sep 21;13(9):815. doi: 10.3390/pathogens13090815 (PMC11435334; doi:10.3390/pathogens13090815)
Supplement: Supplementary file 1 [file pathogens-13-00815-s001.zip › Supplementary_material_S1.pdf]

Supplementary Material S1

DNA quality check of genomic DNA extracted from random blood samples

1. **1% agar gel electrophoresis**

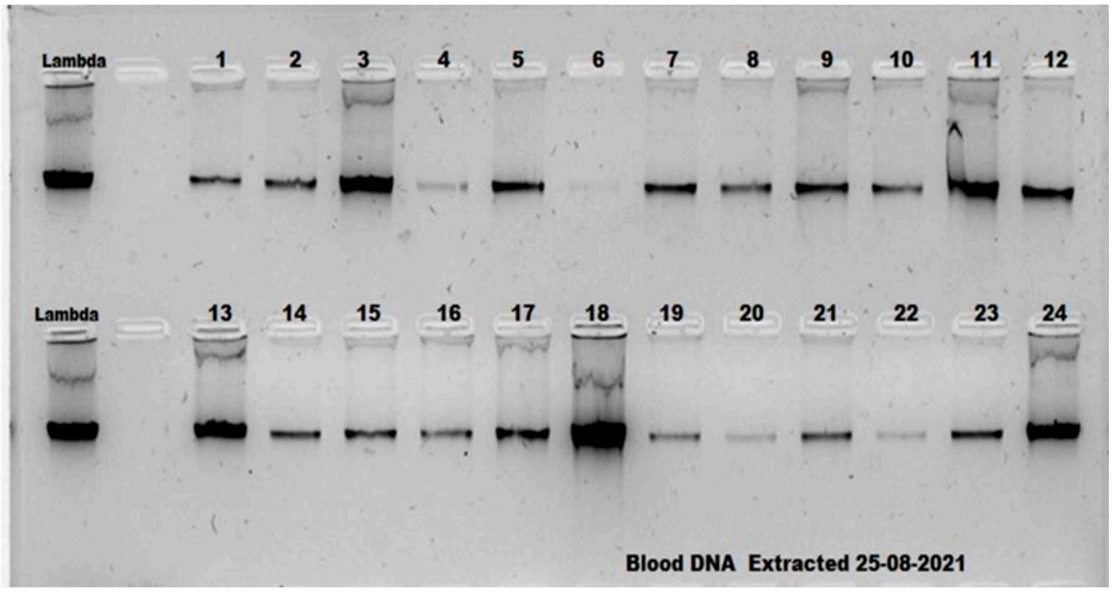

Figure S1. DNA quality check results from 24 random blood DNA samples using 1% agarose gel electrophoresis

Gel electrophoresis was used to check for DNA degradation. If the results of agarose gel electrophoresis show only high molecular weight genomic DNA bands and no other bands or smears, then it means that the DNA sample has not been degraded during the extraction process.

For both rows, the first and second wells were for positive and negative controls respectively. In row 1, sample number had almost no genomic DNA. In the second row, well number 20 and 22 had almost no genomic DNA.

Overall results the genomic DNA were not degraded and can be used for further analysis.

2. **Nanodrop spectrophotometer**

Table S1. Nanodrop quality checks for the DNA from random blood samples extracted using TANBeads® Nucleic Acid Extraction Validation Kit (OptiPure Blood DNA Auto Plate).

Well	Sample ID	Conc.(ng/ul)	A260	A280	A260/A280
**A1**	1	5.737	0.115	0.089	1.3
**A2**	2	14.75	0.295	0.165	1.79
**A3**	3	28.49	0.57	0.319	1.79
**A4**	4	11.93	0.239	0.127	1.88
**A5**	5	5.485	0.11	0.061	1.80
**A6**	6	21.7	2.455	1.792	1.37
**A7**	7	13.55	0.271	0.16	1.69
**A8**	8	58.54	1.171	0.756	1.55
**A9**	9	10.22	0.204	0.112	1.82
**A10**	10	20.04	0.401	0.208	1.93
**A11**	11	12.98	0.16	0.085	1.87
**A12**	12	10.35	0.207	0.108	1.91

For DNA purity estimation, a ratio of absorbance at 260nm/280nm is used, if the ratio is ~1.8, the DNA is generally considered pure. Good-quality DNA will have an A260/A280 ratio of 1.7–2.0. However, a reading of 1.6 still makes DNA suitable for use.

The results of the nanodrop spectrophotometer DNA quality check (**Table 1)**, shows that most of the samples passed the quality check except for sample number 1, 6, and 8 giving an efficiency of 75%.
