# Supplementary material for: Brucella Species Circulating in Smallholder Dairy Cattle in Tanzania
Source: Pathogens. 2024 Sep 21;13(9):815. doi: 10.3390/pathogens13090815 (PMC11435334; doi:10.3390/pathogens13090815)
Supplement: Supplementary file 1 [file pathogens-13-00815-s001.zip › Supplementary_material_S2.pdf]

Supplementary Materials S2

1. Polymerase chain reaction conditions for *Brucella* genus detection

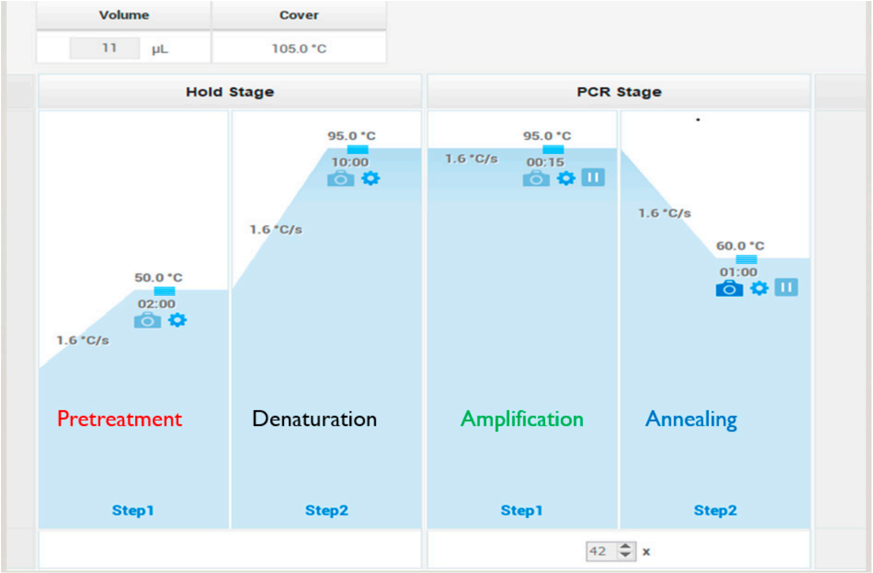

Figure S2. The PCR condition settings used for running the DNA samples in the quantitative real-time PCR machine

2 Limit of detection of the reference material(serial dilutions) for genus detection

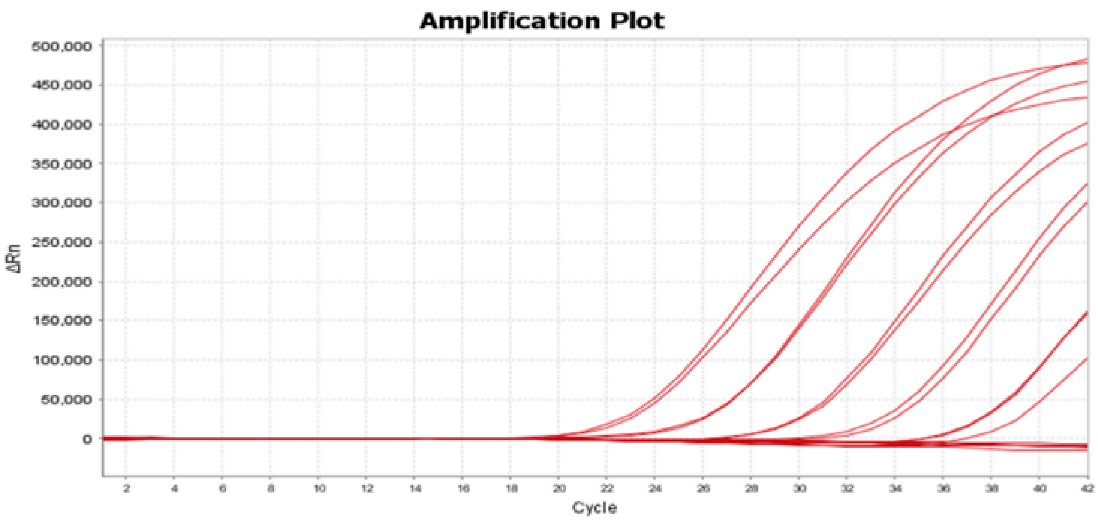

Figure S3. Amplification plot of the serial dilutions of the reference DNA for optimization of the assay for *Brucella* genus detection using *B. abortus* reference material and IS711 primers and probe

3. Standard curve for *Brucella* genus detection

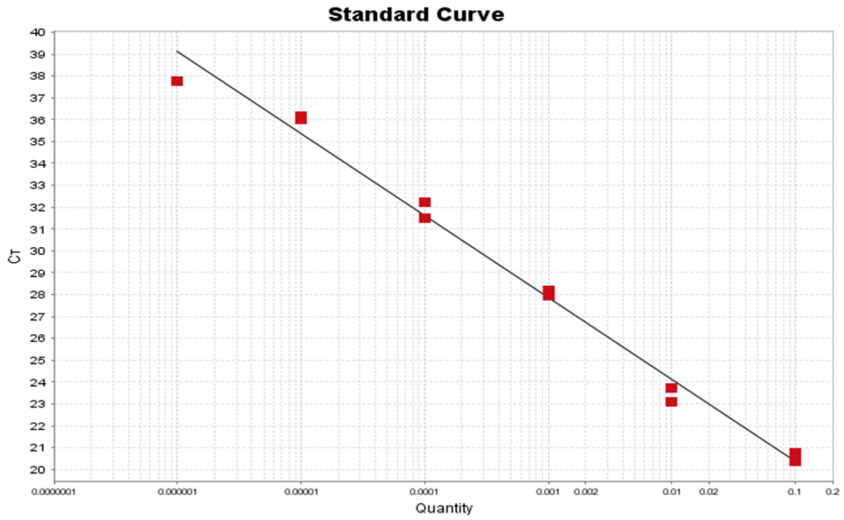

Figure S4. The IS711 standard curve for detection of *Brucella* genus. Efficiency (Eff: 90.55%, R^2: 0.988, slope: -3.56, and y-intercept: 16.63)

The assays for *Brucella* species detection

1. The PCR steps and conditions (Temperature and Time) setting for *Brucella* species detection

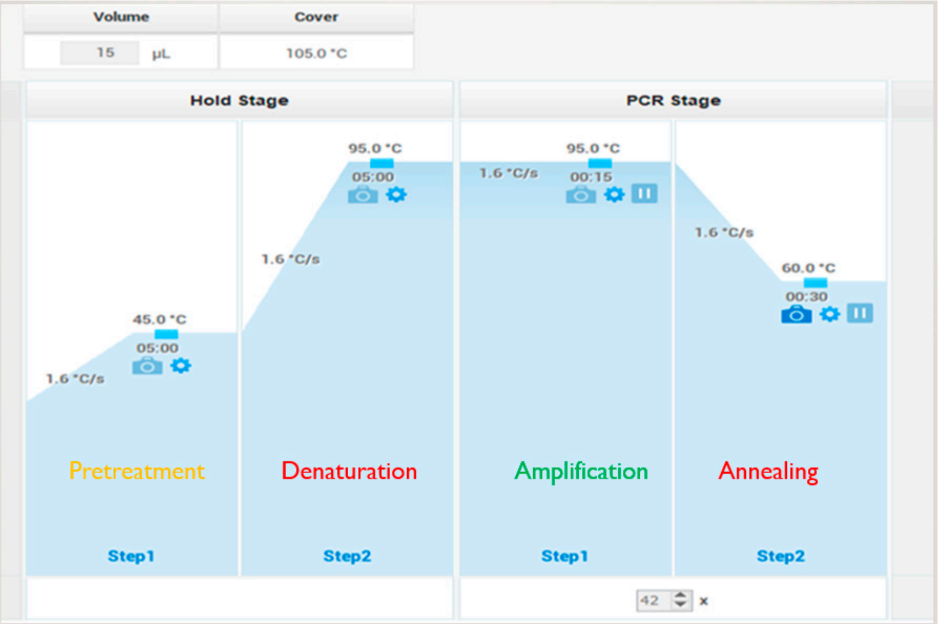

Figure S5. The PCR steps and conditions (Temperature and Time) for the *B. abortus* and *B. melitensis* detection

2. To determine the limit of detection (LOD) for an assay to detect *B. abortus* reference DNA (*B. abortus* 544)

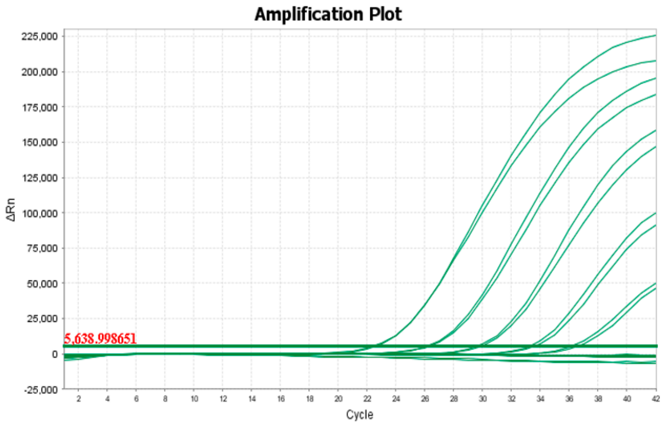

Figure S6. Amplification plot of the serial dilution of the *B. abortus* reference DNA for optimization of the assay for *B. abortus* detection using ***alkB*** primer pair

First curve is 10^{-1} µg/µl and last curve is 10^{-5} µg/µl dilution of the reference material. Therefore 10^{-5}µg/µl dilution of the reference material was used as the limit of detection (the lowest concentration the assay can detect) for *B. abortus* detection

3. The standard curve for *B. abortus* detection

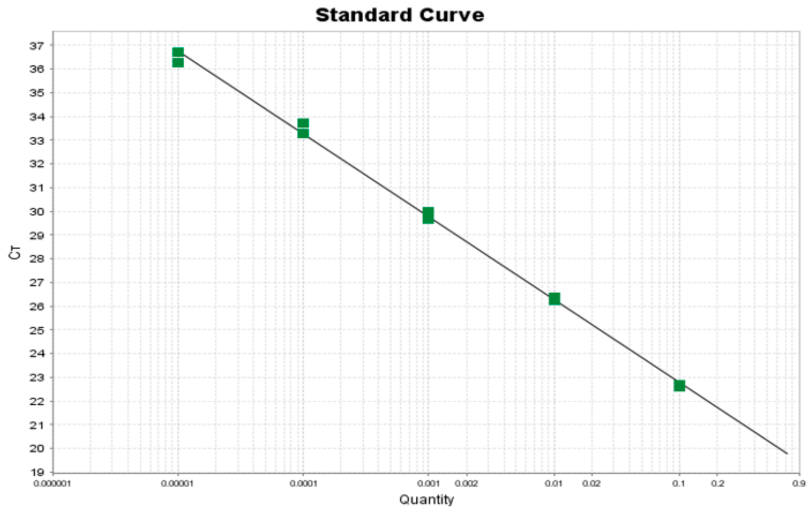

Figure S7. The *alkB* target standard curve for detection of *B. abortus*. Efficiency (Eff: 93.496%, R^2: 0.998, slope: -3.488, and y-intercept: 19.288)

4. To determine the limit of detection (LOD) for an assay to detect *B. melitensis*

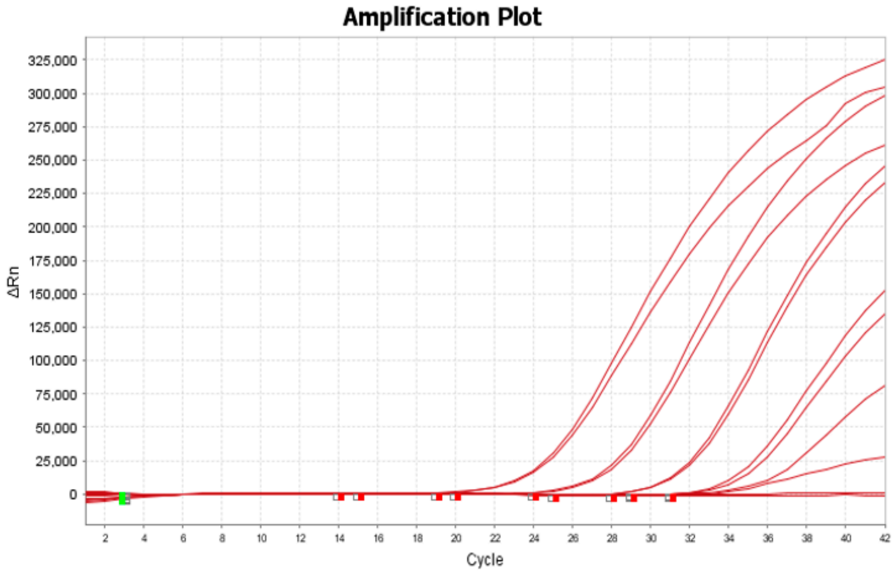

Figure S8. Amplification plot of serial dilutions of *B. melitensis* reference DNA to detect the limit of detection of the assay using BMEI1162 primers

First curve is 10^{-1} µg/µl and last curve is 10^{-5} µg/µl dilution of the reference material. Therefore 10^{-5}µg/µl dilution of the reference material was used as the limit of detection (the lowest concentration the assay can detect) for *B. melitensis* detection.

5. The standard curve for *B. melitensis* detection

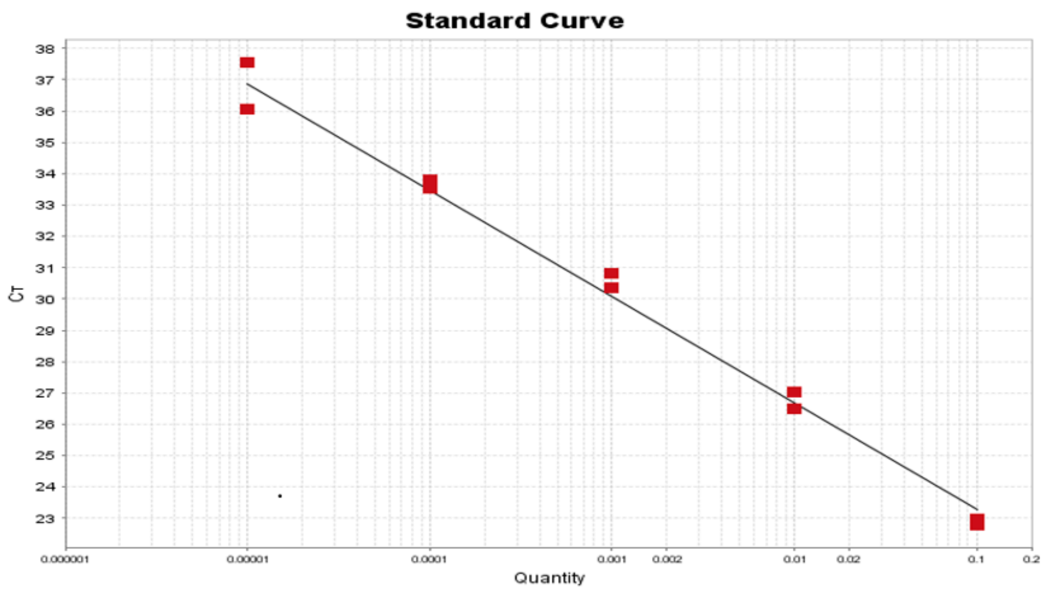

Figure S9. The *BMEI1162* target standard curve for detection of *B. melitensis*. Efficiency (Eff: 97.149%, R^2: 0.984, slope: -3.392, and y-intercept: 19.885)
